# Rvisdiff: An R package for interactive visualization of differential expression

**DOI:** 10.3389/fbinf.2024.1349205

**Published:** 2024-09-02

**Authors:** David Barrios, Carlos Prieto

**Affiliations:** Bioinformatics Service, Nucleus, University of Salamanca, Salamanca, Spain

**Keywords:** expression analysis, RNA-seq, data visualization, protein array, R package

## Abstract

Rvisdiff is an R/Bioconductor package that generates an interactive interface for the interpretation of differential expression results. It creates a local web page that enables the exploration of statistical analysis results through the generation of auto-analytical visualizations. Users can explore the differential expression results and the source expression data interactively in the same view. As input, the package supports the results of popular differential expression packages such as DESeq2, edgeR, and limma. As output, the package generates a local HTML page that can be easily viewed in a web browser. Rvisdiff is freely available at https://bioconductor.org/packages/Rvisdiff/.

## Highlights

- Rvisdiff provides analytical and interactive graphs that enable the comprehensive analysis of differential expression results.
- Graphs are connected with the differential expression table of results and interconnect gene expression with analysis results.
- All the figures are interactive and provide useful features for data exploration.
- Rvisdiff generates a local HTML report, which ensures portability, ease of use, and data privacy. This architecture avoids software and virtual machine installations.

## Introduction

Comparison of RNA expression levels between conditions is a routine analysis that became common in the omics era with the appearance of expression microarrays. These analyses, called differential expression analyses, take a normalized expression matrix as input and perform statistical tests to detect quantitative changes in expression levels between experimental groups. Differential expression analyses generate extensive reports that require manual inspection for optimization and interpretation of the results. At present, there are 362 R packages in the Bioconductor repository under the “Differential Expression” category, which solve tasks such as normalization, batch correction, contrast analysis, interpretation, and visualization. Some visualization techniques are implemented in the most popular differential expression packages as static graphs. For example, MA plots and volcano plots can be represented using the resulting objects from the DESeq and edgeR packages, respectively (Robinson et al., 2010; Love et al., 2014). These graphics are useful for evaluating global analysis, but they do not allow for gene-specific exploration.

Standalone software and R packages have been developed to enhance the visualization and interpretation of differential expression results. Supplementary Table 1 summarizes and compares recent approaches developed for visualization. These approaches have been implemented using various architectures, such as web servers, R packages, HTML, or JavaScript static websites. GLIMMA is a Bioconductor package that generates static web pages with three graph types: scatter plots, point plots, and bar plots (Su et al., 2017). It is used for representing MA plots, volcano plots, and MDS results. DEIVA also represents MA and volcano plots using JavaScript technology, which requires a server for its visualization (Harshbarger et al., 2017). For a wider variety of graphs, the package ViDGER generates nine different visualizations, but the resulting graphs are not interactive (McDermaid et al., 2019). Another R package that generates an HTML report is SARTools; it provides an analysis pipeline, but the final report is not interactive (Varet et al., 2016). The Bioconductor package metaseqR2 also provides a pipeline that enables the comparison of different analysis methods for differential expression and provides static graphs as output (Fanidis and Moulos, 2021). A different approach is the development of a web server as the Degust platform. Users have to upload a matrix of read counts for its analysis, and the platform generates a table of results and interactive graphs. Web server solutions are suitable for non-bioinformatics users, but they have difficulties with input format deviations, automation of analysis tasks, reproducibility, and data security.

We have designed Rvisdiff to integrate graphs into an easy-to-use and interactive web page. Users can explore the differential expression results and the source expression data in the same view.

As input data, the package receives two tables: one with the differential expression results and another with the raw/normalized expression data. It detects the default output of the DESeq2, edgeR, and limma packages, and no data conversion is needed (Smyth, 2005; Robinson et al., 2010; Love et al., 2014). Users can also generate a custom data frame that integrates a statistical test output with fold change and mean calculations for each variable.

As output, the package generates a local HTML page that can be viewed in a web browser. The installation of additional software, such as application servers or programming languages, is not necessary. This feature ensures portability and ease of use. Moreover, the results are stored in the local computer, avoiding any network sharing or data uploads to external servers, which ensures data privacy.

## Results

### Rvisdiff execution

Rvisdiff’s main function is *DEReport*, which generates the graphical interface. It accepts three parameters:

- Differential expression results: receives a data frame with the full output of a differential expression analysis. The function will detect the column names of the main analysis packages and adapt them to the final report. These column names can be specified using specific parameters for each data column.
- Raw count data or normalized expression values: the *count data* and *normalized* parameters specify the number of reads or the normalized expression per gene for each sample, respectively. It is used for the graphical representation of expression values.
- Input conditions: the group parameter specifies the category of each sample in the differential expression analysis. Samples will be labeled with a color code that represents their class.

Once this function is executed, an HTML web page will be generated and can be viewed on a web browser. The file system path of this output page can be specified with the *directory* parameter.

### Output visualization and interoperability

**FIGURE 1:**
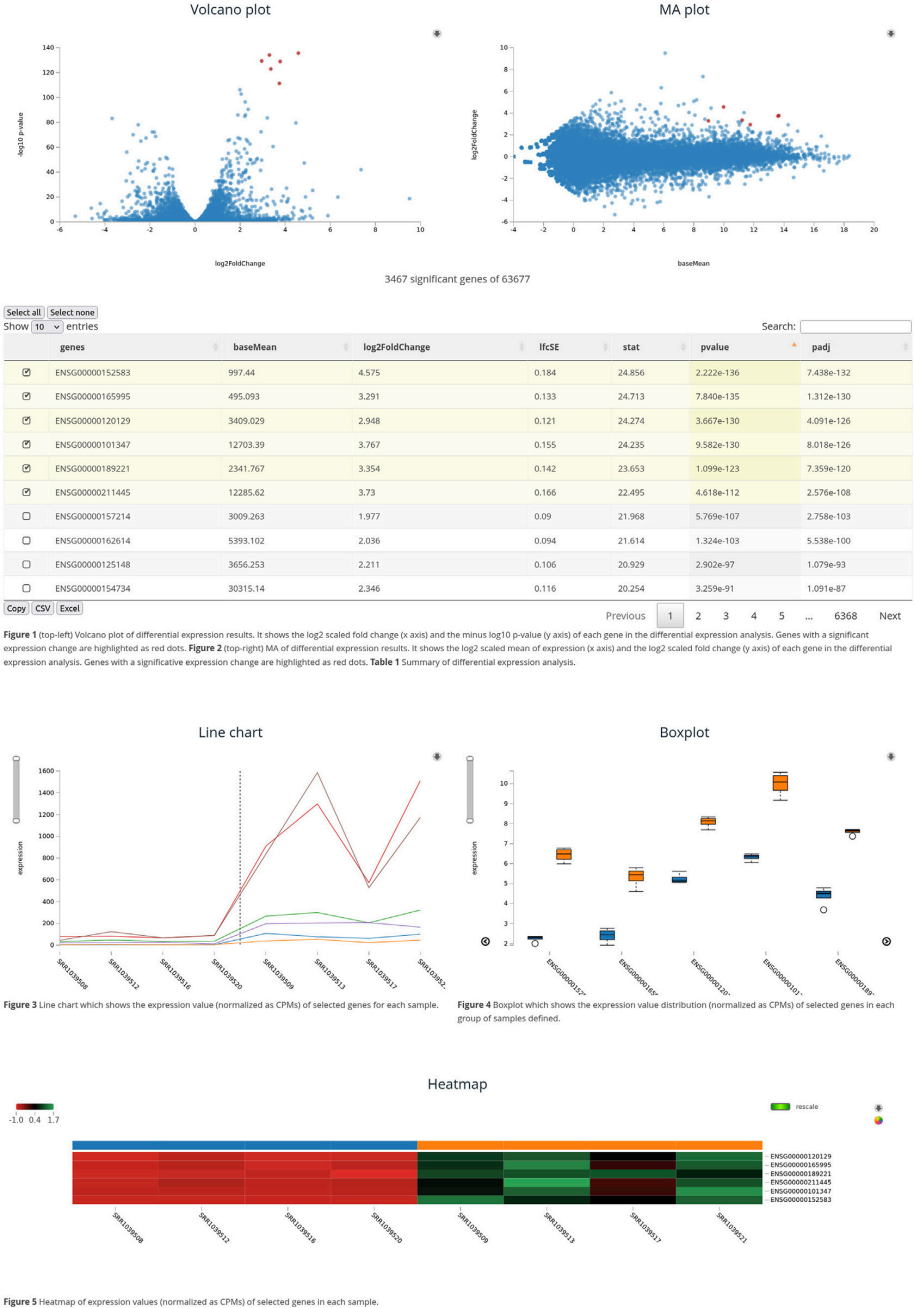
Rvisdiff interface. Chart of the Rvisdiff web interface showing the volcano plot, MA-plot, results table, line diagram, box plot, and heatmap. These graphs are interactive and connect the output of the differential expression analysis and gene expression values.

The graphical user interface has been designed to enable the easy exploration of differential expression results and the visualization of expression values for a set of genes. Figure 1 shows an example of a web page generated by the package. It contains the following graphs:

- Volcano plot: it is represented as a scatter plot that shows the relation between the rate of change (in logarithmic scale) and the resulting p-value (in minus logarithm 10 scale). Points are highlighted when the mouse hovers over the results table, and the variable name appears on mouseover. This interactive feature enables the easy identification of differentially expressed genes and possible false positives.
- MA-plot: it is a scatter plot showing mean expression values versus the rate of change; both are plotted on the logarithmic scale to avoid excessive scatter. It enables the detection of positives with low expression and the difference between over- and underexpressed results. It has the same interactivity features as volcano plots.
- Line diagram: the gene expression levels in each sample are represented as a line. Several lines are represented in the same diagram, showing the expression of different genes. A line divides the graph into two parts, following the input phenotype. The sample name is shown on mouseover, and the graph scale can be changed with the slide controls.
- Box plot: this graph type allows us to visualize the distribution, degree of asymmetry, extreme values, and the value of quartiles. It is also useful for comparing two distributions if we represent them on the same graph. The resulting graphs show the difference in the expression of genes between conditions and identify outliers and different distributions.
- Cluster heatmap: expression data are displayed in a grid where each row represents a gene and each column represents a sample. The color and intensity of the boxes are used to represent changes (usually scaled per gene, avoiding absolute values) in gene expression. The heatmap also shows a clustering tree that groups genes and samples based on the similarity of their gene expression patterns. Users can change the color scale and toggle the rendering from raw to scaled values. Moreover, the graph provides a zoom feature that enables one to focus on a set of samples or genes.

All the graphs are connected to the differential expression table of results. With the user action, points of scatter plots are highlighted, lines are added to the line diagram, boxes appear or disappear on the box plot, and genes represented on the cluster heatmap are selected. These interactive actions, combined with the graphical interactive features, enable the integrative exploration of results that connect differential expression output to gene expression data.

## Materials and methods

### Implementation

Rvisdiff has been developed as an R Package, and it was accepted in the Bioconductor repository. It has been checked against the latest development version of R/Bioconductor and reviewed by an expert in R package development and repository deployments. The package includes vignettes, unit tests, and help files and follows Bioconductor requirements for good development practices. Programming technologies used in the project include R for data handling and data analysis, and jQuery, D3.js, DataTables, HTML5, and CSS3, for the web interface development.

## Discussion

Rvisdiff was designed based on our experience as analysts in a bioinformatics core facility. Agile exploration of results is essential in RNA-Seq analyses because they yield heterogeneous results depending on the chosen methods and parameters. Wet laboratory scientists are the foremost experts on their data, and they should have the ability to query specific gene results. It is important to provide tools that do not require IT skills for their use to ensure proper interpretation. Moreover, data privacy is a key aspect of research and is compromised when raw data or results are uploaded to web servers. For these reasons, we have chosen a static HTML page as output, which meets both of these requirements. However, this format has limitations. For example, users cannot modify analysis parameters or launch new analyses. Actions on the resulting page are limited to the methods provided by JavaScript technologies, which are mainly focused on visualization and event handling. Currently, there is a lack of statistical libraries in JavaScript, making it difficult to develop interactive data analysis methods. However, we anticipate the creation and integration of new libraries into our package to improve interactive statistical calculations.

The next development steps for Rvisdiff will focus on adding controls for graph customization to improve visualization and obtain publication-ready images. Another objective is to improve the integration of our package into current analysis pipelines, Bioconductor tutorials, and reporting tools. This will require the development of new functions and adapting reports for integration. Finally, we will address user suggestions and limitations observed in our usability tests, conduct a viability analysis, and implement the desired features.

## Data Availability

The original contributions presented in the study are included in the article/Supplementary Material; further inquiries can be directed to the corresponding author.
