# Supplementary material for: Rvisdiff: An R package for interactive visualization of differential expression
Source: Front Bioinform. 2024 Sep 2;4:1349205. doi: 10.3389/fbinf.2024.1349205 (PMC11402892; doi:10.3389/fbinf.2024.1349205)
Supplement: Supplementary file 1 [file DataSheet1.pdf]

	Graph Features			Graph Types					Software		
	Interactive[1]	Interconnected[2]	Dynamic[3]	MA-Plot	Volcano Plot	Heatmap	Boxplot	Line Plot	License	Implementation	Data Privacy
**Rvisdiff**	Yes	Yes	Yes	Yes	Yes	Yes	Yes	Yes	GPL-2 | GPL-3	JS - HTML5	Private/HTML file
**Glimma**	Yes	Yes	Yes	Yes	Yes	No	No	No	GPL-3	JS - HTML	Private/HTML file
**Degust**	Yes	Yes	Yes	Yes	Yes	Yes	No	Yes	GPL v3	Web Server	Online Webserver
**DEIVA**	Yes	Yes	No	Yes	Yes	No	No	No	MIT	node.js	Local server
**Vidger**	No	No	No	Yes	Yes	No	Yes	No	GPL-3	R	Private/Image files
**SARTools**	No	No	No	Yes	Yes	No	No	No	GPL-2	HTML	Private/HTML file
**MetaseqR2**	Yes	No	No	Yes	Yes	Yes	Yes	Yes	GPL (>= 3)	JS - HTML	Private/HTML file

Supplementary table S1. Comparison of pipelines, web servers and cloud platforms for the analysis of RNA-Seq data.

[1]Interactive: Users can interact with the graph to change visual aspects or obtain information.

[2]Interconnected: Graphs are connected and changes are coordinated.

[3]Dynamic: Data can be represented and added to the graph upon user request.
